# What factors influence health professionals to use decision aids for Down syndrome prenatal screening?

**DOI:** 10.1186/s12884-016-1053-2

**Published:** 2016-09-05

**Authors:** Johanie Lépine, Maria Esther Leiva Portocarrero, Agathe Delanoë, Hubert Robitaille, Isabelle Lévesque, François Rousseau, Brenda J. Wilson, Anik M. C. Giguère, France Légaré

**Affiliations:** 1 Canada Research Chair in Shared Decision Making and Knowledge Translation and Research Axis of Population Health and Practice-Changing Research, CHU de Québec Research Centre, Quebec, Canada; 2 Department of Obstetrics and Gynecology, Faculty of Medicine, Université Laval, Quebec, Canada; 3 Department of Molecular Biology, Medical Biochemistry and Pathology, Faculty of Medicine, Université Laval, and MSSS/FRQS/CHUQ Research Chair in Health Technology Assessment and Evidence Based Laboratory Medicine, Quebec, Canada; 4 School of Epidemiology, Public Health and Preventive Medicine, University of Ottawa, Ontario, Canada; 5 Quebec Centre of Excellence on Aging, CHU de Québec Research Centre, Quebec, Canada; 6 Department of Family Medicine and Emergency Medicine, Faculty of Medicine, Université Laval, Quebec, Canada; 7 CHU de Québec Research Centre (CRCHUQ), Hôpital Saint-François d’Assise, Université Laval, 10 rue de l’Espinay, Local D6-737, Quebec, QC G1L 3L5 Canada

**Keywords:** Patient decision aids, Health professionals, Obstetrician-gynecologists, Family physicians, Midwives, Prenatal screening, Down syndrome, Shared decision making, Theoretical Domains Framework

## Abstract

**Background:**

Health professionals are expected to engage pregnant women in shared decision making to help them make informed values-based decisions about prenatal screening. Patient decision aids (PtDAs) foster shared decision-making, but are rarely used in this context. Our objective was to identify factors that could influence health professionals to use a PtDA for decisions about prenatal screening for Down syndrome during a clinical pregnancy follow-up.

**Methods:**

We planned to recruit a purposive sample of 45 health professionals (obstetrician-gynecologists, family physicians and midwives) involved in the care of pregnant women in three clinical sites (15 per site). Participating health professionals first watched a video showing two simulated consecutive prenatal follow-up consultations during which a pregnant woman, her partner and a health professional used a PtDA about Down syndrome prenatal screening. Participants were then interviewed about factors that would influence their use of the PtDA. Questions were based on the Theoretical Domains Framework. We performed content analyses of transcribed verbatim interviews.

**Results:**

Out of 42 eligible health professionals approached, 36 agreed to be interviewed (86 % response rate). Of these, 27 were female (75 %), nine were obstetrician-gynecologists (25 %), 15 were family physicians (42 %), and 12 were midwives (33 %), with a mean age of 42.1 ± 11.6 years old. We identified 35 distinct factors reported by 20 % or more participants that were mapped onto 10 of the 12 of the Theoretical Domains Framework domains. The six most frequently mentioned factors influencing use of the PtDA were: 1) a positive appraisal (*n* = 29, 81 %, beliefs about consequences domain); 2) its availability in the office (*n* = 27, 75 %, environmental context and resources domain); 3) colleagues’ approval (*n* = 27, 75 %, social influences domain); 4) time constraints (*n* = 26, 72 %, environmental context and resources domain); 5) finding it a relevant source of information (*n* = 24, 67 %, motivation and goals domain); and 6) not knowing any PtDAs (*n* = 23, 64 %, knowledge domain).

**Conclusions:**

Appraisal, PtDA availability, peer approval, time concerns, evidence and PtDA awareness all affect whether health professionals are likely to use a PtDA to help pregnant women make informed decision about Down syndrome screening. Implementation strategies will need to address these factors.

**Electronic supplementary material:**

The online version of this article (doi:10.1186/s12884-016-1053-2) contains supplementary material, which is available to authorized users.

## Background

The decision about whether or not to take the prenatal test for Down syndrome (DS) is a difficult one [1, 2]. Future parents have to decide in a context of uncertainty where results cannot be predicted or guaranteed [3–5]. The decision requires an understanding of probability data, such as the personal risk of carrying a fetus with DS, and of screening test characteristics such as detection, false-positive and false-negative rates. This sensitive decision can also lead to a further, more sensitive decision (i.e. terminating the pregnancy or preparing for a child with special needs). Moreover, with the increased availability of Non-Invasive Prenatal Testing (NIPT) for women considering DS screening, future parents will need to be well supported in the decision process to work through the strengths and limitations of all the available tests, including the option not to test [6–8].

Health professionals are expected to engage pregnant women in shared decision-making to help them make informed values-based decisions [9–12]. Shared decision-making is an interpersonal and interdependent process in which providers and patients collaborate in making decisions about the patient’s health care. The evidence, the providers’ clinical expertise and the unique attributes of patients and their families all play a part in this decision [13, 14]. Using a patient decision aid (PtDA) is one effective way to foster shared decision-making in clinical practice [15, 16]. A systematic review of 115 trials of PtDAs has demonstrated their efficacy: they increase patients’ knowledge scores, their risk perception, the match between their values and choices, and they help patients who are undecided to make up their minds [17]. Despite their proven effectiveness, the widespread adoption of PtDAs has not yet been achieved [18, 19]. In addition, few PtDAs for the decision about DS screening have been identified, and none of them met all the 16 minimum criteria established by the International Patient Decision Aids Standards (IPDAS) [20, 21]. To address possible reasons for low PtDA uptake among various health professionals and to provide information for strategies to implement PtDAs for the decision about DS prenatal screening, we sought to identify factors influencing health professionals’ use of a PtDA about prenatal screening for DS during a clinical pregnancy follow-up. This qualitative study will serve as a first step to developing a theory-based survey questionnaire to quantitatively identify the determinants of health professionals’ intention to use a PtDA for DS screening decisions in preparation for an implementation intervention to promote its optimal use in routine clinical practice.

## Methods

### Study design and context

This qualitative study was embedded in a sequential exploratory mixed-methods study aiming to implement the use of a PtDA to foster shared decision-making in the context of prenatal screening for DS. The overall study is part of the PEGASUS project (PErsonalized Genomics for prenatal Aneuploidy Screening USing maternal blood, Canada). Ethics approval was obtained from the research ethics boards of the Centre de Santé et de Services Sociaux de la Vieille-Capitale (#2013-2014-29) and the CHU de Québec (#B14-02-1929).

### Participants and recruitment

Prenatal care in the province of Quebec, Canada, is offered by obstetrician-gynecologists (about 51 % of pregnancies), family physicians (about 46 %) or midwives (about 3 %) [22]. We wanted to maximize the diversity of perspectives by drawing from clinical sites representing different team approaches to prenatal care and different clienteles. According to Godin et al., for this kind of qualitative study, a minimal sample of 25 to 30 participants is recommended to identify all relevant opinions [23]. We thus aimed to recruit a convenience sample of 45 participants with 15 participants in each professional category. We recruited health professionals from three health centers in the Quebec City area, Canada: obstetrician-gynecologists in a university hospital, family physicians in a family practice teaching unit and midwives in a birthing center.

We included health professionals who were: a) involved in prenatal care; b) family physicians, midwives, obstetrician-gynecologists or interns in these professions; and c) working in any of the three health centers targeted. We excluded health professionals who were on parental or sick leave.

The project coordinator and a research assistant first met the family physicians, the midwives and the obstetrician-gynecologists during a regular professional meeting to explain the study process and secure their collaboration. A research assistant was then assigned to each site to generate a list of health professionals likely to be eligible based on their affiliations, functions and employment status. The research assistant then approached these health professionals, explained the project briefly, assured them that data would be anonymous and confidential, confirmed their eligibility, and invited them to participate. Each participant then signed a consent form and agreed to an interview appointment over the following ten business days.

### Data collection

Participants were interviewed either at their health center or on the phone, depending on their preferences and availability. Before the interview, participants watched a 10-min video showing two simulated consecutive prenatal follow-up consultations between a health professional and a pregnant women and her partner. During the first visit, the health professional gives a PtDA to the couple, explains its purpose, reviews its content and explains the risks and benefits of doing or not doing the prenatal testing. At this point the video presents a close-up of the content of the PtDA page by page and item by item. However, the interviewed health professionals were not offered a printed copy of the decision aid to look at. The PtDA presented in the video had been developed earlier by the team based on an existing decision support tool [24]. Then the health professional invites the couple to look over the PtDA at home. The health professional clarifies that the pregnant women is facing the decision to undergo prenatal screening for DS and that more information can be found in the PtDA that will help the decision-making process. In the second visit (4 weeks later), the pregnant woman, still accompanied by her partner, discusses the decision with the health professional and says she has made her choice. However, the viewer does not find out what she has chosen so that participants would not be influenced by her choice. Immediately after watching the video, to identify factors influencing the use of a PtDA for DS screening during a clinical pregnancy follow-up, semi-structured interviews took place consisting of 11 open-ended questions based on the Theoretical Domains Framework: 1) knowledge/awareness, 2) advantages, 3) disadvantages, 4) incentives, 5) emotions, 6) anticipated regret, 7) social approval, 8) social disapproval, 9) appraisal/evaluation, 10) facilitators and 11) barriers [25].

The Theoretical Domains Framework is comprised of 12 theoretical domains relevant to behavioral change [25–27]. It postulates that factors influencing behavior change can be mapped onto these theoretical domains and can be used to design effective theory-based implementation interventions. In this study, the behavior of interest was defined as follows: use (action) of a PtDA about prenatal screening test for DS (target) in the context of a prenatal care follow-up with a health professional (context) [28]. Time was not specified, as the behavior was hypothetical. At the end of the interview, we also assessed participants’ socio-demographic characteristics. Interviews were audio-recorded and transcribed verbatim. Each recording had an identification code and was uploaded to our database.

### Data analysis

To identify participants’ salient beliefs (henceforth referred to as “opinions”), the second author (MELP) analyzed the full transcripts using a content analysis approach. Then, using N-Vivo v.10 software (QSR International, Melbourne, Australia), similar opinions were grouped into themes (henceforth referred to as “influential factors”) which were mapped onto the Theoretical Domains Framework constructs and then onto 10 of its 12 domains: 1) beliefs about consequences, 2) environmental context and resources, 3) social influences, 4) social/professional role and identity, 5) knowledge, 6) emotions, 7) beliefs about capabilities, 8) motivation and goals, 9) skills, and 10) behavioral regulation. No influential factors mapped onto the two remaining theoretical domains, which are 11) memory, attention and decision processes, and 12) nature of the behaviors. These domains were less relevant to our participants, probably because they had hardly ever used PtDAs in the clinic before [25]. The first author (JL) independently read all the transcripts and double-checked the tree-node. She suggested the addition of relevant categories or themes and the removal of less relevant ones, and discussed a final coding scheme with the second author. Any discrepancies were resolved through discussion with team members. The same procedure was undertaken with all identified influential factors. The number of participants who identified the same influential factor, along with the percentage rate, was calculated for each health professional category (n, %, Table 2) and for all professional categories together (N, %, Table 2). Modal beliefs were also identified (superscripted "a" in Table 2), i.e. the most frequently reported influential factors for each construct (up to 75 %), to give a sense of the relative importance of this factor (some factors, for example, were mentioned more than once by a single participant) [23]. The frequencies of modal beliefs were calculated by summing the total number of quotes for each influential factor (Q) as well as the percentage of times that the same opinion was repeated per construct (%QC) (Table 2).

## Results

### Participant characteristics

**Fig. 1:**
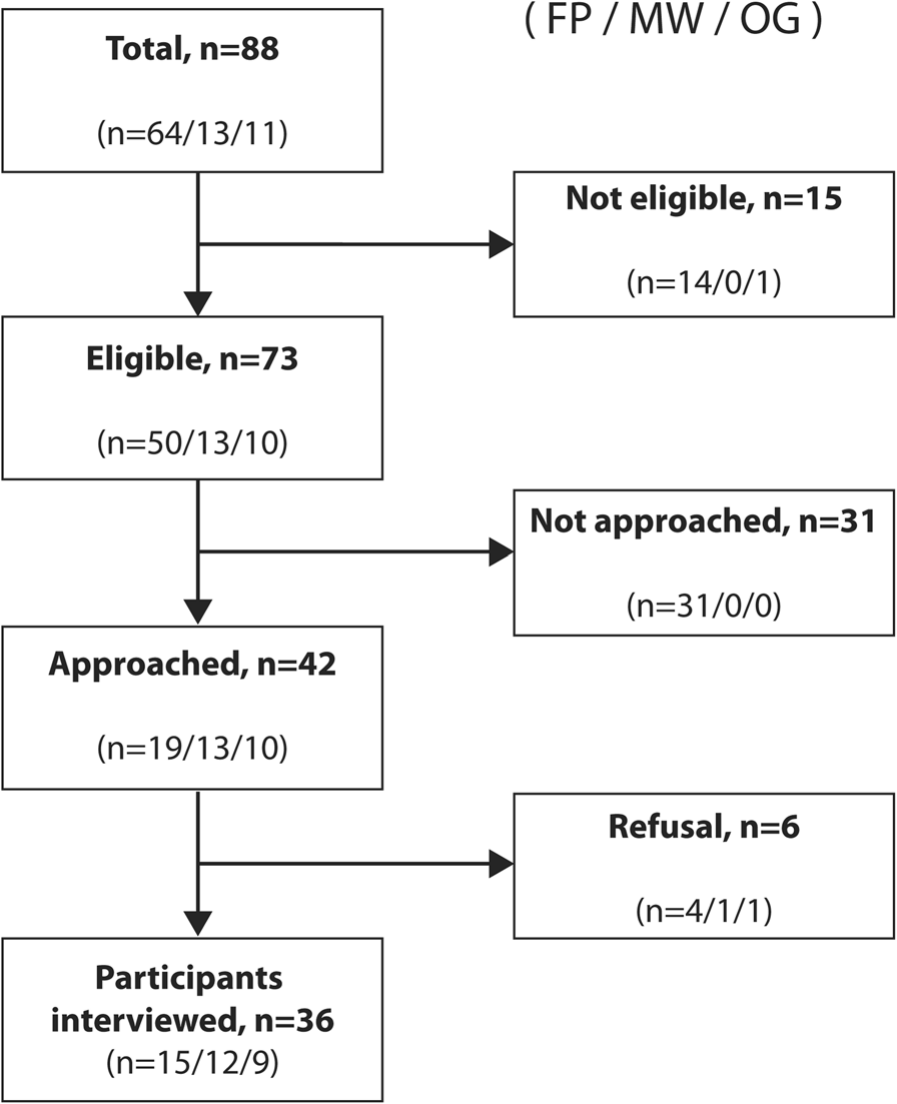
Flow of the participants. *FP* family physicians, *MW* midwives; and *OG* obstetrician-gynecologists

Out of 73 eligible health professionals, 42 were approached between July 25, 2014 and February 28, 2015, and 36 agreed to complete interviews (86 % overall participation rate), including 15 family physicians (79 %), 12 midwives (92 %) and nine obstetrician-gynecologists (90 %). The remaining 31 eligible family physicians were not approached because we stopped recruitment once we had reached the expected sample size for this category (*n* = 15, Fig. 1). Twenty-seven women (75 %) and nine men (25 %) were recruited; four participants were interns (11 %, Table 1); and two obstetrician-gynecologists were interviewed by phone (6 %) instead of in person (*n* = 34, 94 %, data not shown). The mean age was 42.1 ± 11.6 years old, mean practice experience was 12.6 ± 12.0 years, and mean number of prenatal visits per week was 17.4 ± 21.5. The mean length of interviews including video viewing (10 min) was 28 ± 6 min (Table 1).

**Table 1:** Health professionals’ characteristics

Characteristics	FP *n* = 15	MW *n* = 12	OG *n* = 9	Total *N* = 36
Age (years)^a^	40.5 ± 13.4	39.8 ± 8.0	48.1 ± 11.7	42.1 ± 11.6
Sex (n women/men)	10/5	12/0	5/4	27/9
Experience (years)^a^	13.0 ± 12.7	7.7 ± 5.5	18.3 ± 15.2	12.6 ± 12.0
Prenatal visits/week^a^	5.4 ± 5.4	13.6 ± 4.2	42.6 ± 30.5	17.4 ± 21.5
Internship student (n)	3	0	1	4
Interview lengths (min)^ab^	26 ± 6	31 ± 6	27 ± 3	28 ± 6

*FP* family physicians, *MW* midwives; and *OG* obstetrician-gynecologists

^a^Mean ± SD

^b^Interview lengths include watching the video (10 min.)

### Influential factors

A total of 64 influential factors (see Additional file 1) were reported by participating health professionals regarding the use of a PtDA for deciding about prenatal screening for DS during a pregnancy follow-up visit, including 35 factors that were reported by 20 % or more participants in any health professional category (Table 2).

**Table 2:** Frequency of most prevalent influential factors reported by each category of health professional and overall

TDF domains	Constructs	Influential factors (n ≥ 20 %)	#IF	FP *n* = 15 n (%)	MW *n* = 12 n (%)	OG *n* = 9 n (%)	Total *N* = 36 N (%)	Total quote Q (%QC)
Beliefs about consequences	Advantages	It helps patients to think about the decision	1	8 (53)	7 (58)	3 (33)	18 (50)	25 (44)^a^
		Its visual content is helpful for patients	2	5 (33)	4 (33)	2 (22)	11 (31)	14 (25)^a^
		It enables expression of preferences	3	2 (13)	5 (42)	1 (11)	8 (22)	9 (16)
		It promotes decision making	4	1 (7)	4 (33)	3 (33)	8 (22)	9 (16)
	Disadvantages	Its use didn’t fit in with program timing	5	1 (7)	5 (42)	7 (78)	13 (36)	15 (45)^a^
		Its content is incomplete	6	2 (13)	2 (17)	4 (44)	8 (22)	10 (30)^a^
	Anticipated regret	I would regret if I didn’t use it	7	5 (33)	8 (67)	3 (33)	16 (44)	16 (62)^a^
		I would not regret if I didn’t use it	8	4 (27)	1 (8)	5 (56)	10 (28)	10 (38)^a^
	Appraisal	Positive appraisal	9	11 (73)	12 (100)	6 (67)	29 (81)	33 (92)^a^
		Negative appraisal	10	0 (0)	1 (8)	2 (22)	3 (8)	3 (8)
Environmental context and resources	Facilitators	Its availability in the office	11	12 (80)	9 (75)	6 (67)	27 (75)	47 (67)^a^
		Its comprehensibility for patients	12	3 (20)	5 (42)	1 (11)	9 (25)	10 (14)^a^
		It must be brief	13	2 (13)	2 (17)	2 (22)	6 (17)	7 (10)
		I have enough time to present it	14	1 (7)	3 (25)	0 (0)	4 (11)	4 (6)
	Barriers	Not having enough time to present it	15	12 (80)	7 (58)	7 (78)	26 (72)	48 (58)^a^
		If it is too complex for patients	16	5 (33)	7 (58)	4 (44)	16 (44)	21 (25)^a^
		If it is available in print form only	17	3 (20)	0 (0)	0 (0)	3 (8)	4 (5)
		If its content is unbalanced/biased	18	0 (0)	3 (25)	0 (0)	3 (8)	3 (4)
Social influences	Approve	Colleagues	19	11 (73)	11 (92)	5 (56)	27 (75)	29 (64)^a^
		My family	20	3 (20)	3 (25)	0 (0)	6 (17)	7 (16)^a^
	Disapprove	Colleagues	21	5 (33)	4 (33)	2 (22)	11 (31)	11 (58)^a^
		A colleague with extensive experience	22	3 (20)	0 (0)	0 (0)	3 (8)	3 (16)^a^
Social/professional role and identity	Moral norms	It is my duty to present it	23	2 (13)	3 (25)	2 (22)	7 (19)	8 (67)^a^
Knowledge	Knowledge	I don’t know of any PtDAs	24	11 (73)	4 (33)	8 (89)	23 (64)	23 (59)^a^
		I know the government pamphlet	25	1 (7)	9 (75)	0 (0)	10 (28)	10 (26)^a^
		I know a PtDA for another decision	26	4 (27)	1 (8)	1 (11)	6 (17)	6 (15)
Emotions	Emotions	Give me satisfaction	27	2 (13)	2 (17)	4 (44)	8 (22)	9 (53)^a^
		Reassure me	28	3 (20)	1 (8)	0 (0)	4 (11)	4 (24)^a^
Beliefs about capabilities	Self-efficacy	I feel comfortable to use it	29	3 (20)	7 (58)	1 (11)	11 (31)	13 (68)^a^
Motivation and goals	Incentives	It is a relevant source of information	30	11 (73)	9 (75)	4 (44)	24 (67)	38 (75)^a^
		If I have to decide with my patient	31	1 (7)	6 (50)	3 (33)	10 (28)	11 (22)
		My patient’s uncertainty	32	0 (0)	0 (0)	2 (22)	2 (6)	2 (4)
Skills	Skills development	Need a prior training to use it properly	33	4 (27)	4 (33)	1 (11)	9 (25)	11 (100)^a^
Behavioral regulation	Action planning	Its prior presentation by a nurse	34	8 (53)	0 (0)	3 (33)	11 (31)	17 (57)^a^
		Given to patient before consultation	35	2 (13)	0 (0)	5 (56)	7 (19)	13 (43)^a^

*TDF* Theoretical Domains Framework, *#IF* Influential factor number (see Table 3), *FP* family physicians, *MW* midwives, *OG* obstetrician-gynecologists. *Q* Number of quotes for each influential factor , *%QC* Percentage of times that each influential factor was repeated per construct

^a^modal beliefs

### Beliefs about consequences domain

This domain has four theoretical constructs: advantages, disadvantages, anticipated regret and appraisal. The two most frequently reported advantages of using a PtDA for decisions about prenatal screening for DS were: 1) it helps patients to think about the decision [*N* = 18(50 %); Q = 25(44 %)^a^]; and 2) its visual content is helpful for patients [*N* = 11(31 %); Q = 14(25 %)^a^]. Obstetrician-gynecologists reported these two advantages less frequently (*n* = 3, 33 % and *n* = 2, 22 %, respectively) than did the other two professionals. Five midwives (*n* = 5, 42 %) said that an advantage of the PtDA was that it enables patients to express their preferences, while only one obstetrician-gynecologist (*n* = 1, 11 %) and two family-physicians (*n* = 2, 13 %) reported this. Four midwives (*n* = 4, 33 %) and three obstetrician-gynecologists (*n* = 3, 33 %) thought that another advantage was that PtDAs promote decision making, but only one family physician thought this (*n* = 1, 7 %, Table 2).

The most frequently reported disadvantage was that PtDA use did not fit in with the timing of the current DS prenatal screening program covered by the government of the province of Quebec [*N* = 13(36 %); Q = 15(45 %)^a^]. While the video proposes 4 weeks for thinking about the decision between the first prenatal visit and the second, pregnant women may actually have less than 3 weeks between the first visit, generally between 10 and 12 weeks of pregnancy, and the first blood sample for DS screening, which must be between 10 and 13 weeks of pregnancy. While only one family physician mentioned this (*n* = 1, 7 %), five midwives (*n* = 5, 42 %) and seven obstetrician-gynecologists (*n* = 7, 78 %) mentioned this factor. The latter also said more frequently that the PtDA content is incomplete (*n* = 4, 44 %, Table 2).

Overall, for the anticipated regret construct, more health professionals said they would regret it if they did not use a PtDA [*N* = 16(44 %); Q = 16(62 %)^a^] compared to those who said they would not regret it [*N* = 10(28 %); Q = 10(38 %)^a^]. Almost all midwives mentioned they would regret not using it (*n* = 8, 67 %), while family physicians (*n* = 5, 33 %) and obstetrician-gynecologists (*n* = 3, 33 %) were less likely to regret not using it (Table 2).

For the appraisal construct, the majority of health professionals appraised the use of a PtDA positively, i.e. thought its use would be good, useful, beneficial, or excellent [*N* = 29(81 %); Q = 33(92 %)^a^], especially midwives (*n* = 12, 100 %) (Table 2).

### Environmental context and resources domain

This domain has two constructs: facilitators and barriers. The most frequently reported facilitator was its availability in the office [*N* = 27(75 %); Q = 47(67 %)^a^]. Midwives mentioned comprehensibility for patients more frequently than the others (*n* = 5, 42 %) and a quarter mentioned having enough time to present it as a facilitator (*n* = 3, 25 %, Table 2).

The two most frequently reported barriers were: 1) not having enough time to present it during a clinical encounter [*N* = 26(72 %); Q = 48(58 %)^a^]; and 2) if it is too complex for patients [*N* = 16(44 %); Q = 21(25 %)^a^]. Some family physicians considered PtDAs in print form only as a barrier (*n* = 3, 20 %) while some midwives considered unbalanced/biased PtDAs as a barrier (*n* = 3, 25 %, Table 2).

### Social influences domain

This domain has two opposite constructs: people who would approve and people who would disapprove. Health professionals most frequently reported their colleagues as being both the people who would approve of their using a PtDA [*N* = 27(75 %); Q = 29(64 %)^a^], and to a lesser extent, the people who would disapprove of it [*N* = 11(31 %); Q = 11(58 %)^a^]. No obstetrician-gynecologists reported their family as a social influence (Table 2), and only a few health professionals said that their patients/clients would approve [*N* = 5(14 %); Q = 5(11 %), see Additional file 1] or disapprove of PtDA use [*N* = 2(6 %); Q = 2(11 %), see Additional file 1].

### Social/professional role and identity domain

This domain has one construct: moral norms. The most frequently reported factor relating to moral norms was that seven health professionals felt it was their duty to present the PtDA to their patients [*N* = 7(19 %); Q = 8(67 %)^a^] (Table 2).

### Knowledge domain

The majority of health professionals said they did not know of any PtDAs [*N* = 23(64 %); Q = 23(59 %)^a^] although some said they knew about the government pamphlet (not a decision aid [21]) on the prenatal screening program covered by the province of Quebec [*N* = 10(28 %); Q = 10(26 %)^a^] [29]. Some participants reported that they knew of PtDAs for other decisions (prostate cancer, breast cancer, and respiratory tract infections) [*N* = 6(17 %); Q = 6(15 %)]. More midwives than other health professionals were aware of the existence of PtDAs (only four said they did not know of any PtDAs, *n* = 4, 33 %) and of the government pamphlet (*n* = 9, 75 %) about prenatal screening for DS. Family physicians appeared to be more familiar with PtDAs for other decisions (*n* = 4, 27 %, Table 2).

### Emotions domain

The most frequently reported emotion regarding the use of a PtDA was that its use would give satisfaction [*N* = 8(22 %); Q = 9(53 %)^a^], especially among obstetrician-gynecologists (*n* = 4, 44 %, Table 2).

### Beliefs about capabilities domain

This domain has one construct: self-efficacy. The most frequently reported factor about self-efficacy was that health professionals would feel comfortable using it [*N* = 11(31 %); Q = 13(68 %)^a^], especially midwives (*n* = 7, 58 %, Table 2).

### Motivation and goals domain

This domain has one construct: incentives. The most frequently reported incentive was that health professionals would use the PtDA because it is a relevant source of information [*N* = 24(67 %); Q = 38(75 %)^a^]. However, fewer obstetrician-gynecologists thought so (*n* = 4, 44 %). Compared to the others, fewer family physicians mentioned the incentive of having to decide with their patients (*n* = 1, 7 %, Table 2).

### Skills domain

Health professionals reported only one influential factor in the skills development construct. Overall, a quarter of the health professionals said they would need prior training to use the PtDA properly during a clinical encounter [*N* = 9(25 %); Q = 11(100 %)^a^], while fewer obstetrician-gynecologists thought so (*n* = 1, 11 %, Table 2).

### Behavioral regulation domain

This domain has one construct: action planning. The two most frequently reported preliminary steps were that the PtDA could be explained by a nurse before the consultation [*N* = 11(31 %); Q = 17(57 %)^a^], followed by that the PtDA could be given to patients before the consultation [*N* = 7(19 %); Q = 13(43 %)^a^]. None of the midwives reported these two factors (Table 2).

Table 3 presents examples of opinions (translated from the French originals) by participants that illustrate each of the 35 influential factors (IF) listed in Table 2.

**Table 3:** Examples of opinions that illustrate each influential factors

#IF	Illustrative opinions (translated from the French originals)
1	*It forces the patients to think a little. (OG)*
2	*A visual aid is certainly a tool that helps people understand things better. (OG)*
3	*The fact that they can write what matters most to them, it is a good process. (MW)*
4	*We make sure that patient has really given informed consent. (OG)*
5	*I don’t have time to give it to women and wait for them to come back, it will be too late. (OG)*
6	*There’s just information about the public screening, but I also talk about private tests. (OG)*
7	*I would feel bad if I didn’t use it. (MW)*
8	*I wouldn’t feel guilty for not using it. (OG)*
9	*I would find it good. I think that it is a good tool. (FP)*
10	*There are people who think that it is not useful. (OG)*
11	*It would help to have a bunch of copies. (OG)*
12	*It must be simple and easy to use. (FP)*
13	*Anyway, it must be short and sweet. (MW)*
14	*We have one and a half hours for the first visit, so we have time to present it. (MW)*
15	*The time we have is a disadvantage. (OG)*
16	*I would not use it if it is too confusing. (MW)*
17	*Our patients tell us: You gave me too many documents to read, I didn’t read it. (FP)*
18	*If I have the feeling that the tool promotes one choice, I would feel uncomfortable using it (MW)*
19	*The other midwives. (MW)*
20	*I have children, who have children, and I think they would approve of my using it. (FP)*
21	*I would say probably the team. (FP)*
22	*Those who have more experience could dissuade me. (FP)*
23	*I have no choice but to give it to patients. (FP)*
24	*Not at all! (FP)*
25	*I know this pamphlet. (MW)*
26	*For prostate cancer, with the little cartoons. (FP)*
27	*It would be satisfaction. (MW)*
28	*It would reassure me, because I have something that proves what I’m telling them (FP)*
29	*I would feel comfortable to use it. (FP)*
30	*It is interesting to have evidence about the problem. (FP)*
31	*I would use it to help me to help them to make a decision. (MW)*
32	*I would give the tool to a couple who is really undecided. (OG)*
33	*To train us how to transmit it to couples. (MW)*
34	*One part could be done by the nurse beforehand and I could do the other part. (OG)*
35	*Maybe if patients have it in advance, and have done a first reading before meeting us. (FP)*

*#IF* Influential factor number (see Table 2). *FP* family physicians, *MW* midwives; and *OG* obstetrician-gynecologists

## Discussion

In this qualitative study, we elicited factors influencing the use of a PtDA in the context of prenatal screening for DS by family physicians, midwives and obstetrician-gynecologists, three categories of health professional involved in prenatal care. Six influential factors were reported by a total of 60 % or more participants: 1) a positive appraisal of PtDA use, 2) its availability in their offices, 3) their colleagues’ approval of them using it, 4) not having enough time to present it, 5) it is a relevant source of information for them and their patients, and 6) not knowing about PtDAs (in decreasing order of frequency). These results lead us to make four observations.

First, all six of the most-reported influential factors in our study have previously been reported about the use of other PtDAs by diverse health professionals (i.e. respirologists, family physicians, gynecologists, geriatricians, surgeons, oncologists). A highly positive appraisal of three PtDAs was reported by Graham et al. in 2007 and to a lesser extent in 2003 [30, 31]. The easy availability of PtDAs as a facilitator has not been clearly reported elsewhere, but their lack of availability has been noted as a disadvantage or barrier, which amounts to the same thing [30, 32]. Graham et al. also identified colleagues’ approval as an important influential factor [30, 31]. Surprisingly, very few health professionals in our study mention their patients as a social influence (approval or disapproval) on their use of a PtDA, despite the fact that PtDAs are designed for them. In our study, the most frequent barrier reported about the use of a PtDA was the lack of time to present it. This barrier is widely reported in the literature about PtDAs and especially about shared decision-making implementation [19, 30–33]. The belief that PtDA is a relevant source of information for both clinicians and the patients is also supported in other studies [30, 31]. Finally, a lack of knowledge/awareness of PtDAs among health professionals, our sixth most influential factor, is supported by Brace et al. [32]. Any implementation strategy for use of PtDAs about DS screening in the clinic needs to specifically consider these six most important factors, a finding consistent with results of previous studies.

Second, some influential factors had different frequencies depending on the category of health professional. Family physicians appeared to be more familiar with PtDAs for other decisions. More midwives were aware of the existence of PtDAs and were more likely than the others to feel that a PtDA could encourage patients to express their preferences. Midwives also felt they would be more comfortable using it, and they did not report that prior presentation/handing out of the PtDA could be a useful preliminary step. The attitude of obstetrician-gynecologists regarding PtDA use was less positive overall: they reported advantages and social approval less frequently and disadvantages more frequently, and fewer predicted they would regret it if they did not use it. Similarly divergent factors among eight types of healthcare providers regarding the intention to engage in an shared decision-making approach in home care have been observed elsewhere [34]. These findings suggest that strategies to implement PtDAs for DS screening need to be tailored to different types of health professionals.

Third, the most frequent barrier and fourth most frequently reported influential factor overall was not having enough time to present a PtDA. This is the most widely reported barrier to implementing shared decision-making in clinical practice across numerous cultural and organizational contexts [19]. Although one study suggested that longer consultations were associated with higher shared decision-making scores on the OPTION scale [35], there is no consistent evidence to support that more time is required for shared decision-making than for conventional care [17]. Interestingly, midwives in our study appeared to dedicate more time to their clients for each prenatal visit (an hour and a half for the first visit) than the other two professionals and they also dedicated 117 % more time to the interviews (Table 1). However, midwives were slightly less likely to mention time concerns. Future programs implementing PtDA use must address this perceived time constraint. Rather than adding yet another task to the health professional’s workload, a PtDA could help them replace their consultation style with an shared decision-making approach, especially if patients have a chance to read the PtDA before the consultation and could themselves become advocates for shared decision-making.

Lastly, there is a great need to publicize the existence and proven benefits of those PtDAs that have been validated and rigorously assessed. PtDAs about DS screening, as well as the Quebec government pamphlet [29], were almost unknown to our participants, especially for family physicians and obstetricians-gynecologists. A study in the context of cancer treatment found a similar proportion of respondents who said the main barrier to their using PtDAs was lack of awareness of their existence [32]. This issue could be addressed by opinion leaders endorsing their use [36]. Governments (such as Quebec’s) that have developed information about DS screening could publicize it better [29].

This study has limitations. First, we recruited health professionals in the Quebec City area. This could limit generalization to Canada and to other countries. Secondly, we did not reach our targeted sample of 15 participants for midwives (*n* = 12) and obstetrician-gynecologist (*n* = 9), because only 13 midwives and 11 obstetrician-gynecologists were eligible in their respective recruitment sites. However, we observed influential factors saturation, or redundancy, at *n* = 13/15 for family physicians, *n* = 8/12 for midwives and *n* = 7/9 for obstetrician-gynecologists, and thus consider we collected all the most prevalent factors. Third, the video shown just prior to the interviews presented a female family physician meeting a couple in a family practice teaching unit. Males, obstetrician-gynecologists and midwives might therefore have felt their situation and concerns were unrepresented; while family physicians might have been tempted to compare their practice with the one depicted in the video. Such feelings may have influenced health professionals’ identification of influential factors. However, we made every effort to mitigate this potential limitation by making the video as relevant as possible to all categories of health professional. We also followed a validated processthat has been successfully used pre viously [37]. The fourth limitation was that our interview grid had only 11 open ended questions. It is possible that adding more questions could have identified more factors [26, 38]. However, we made sure to cover all relevant domains (10 out of 12) and our formula of open-ended questions allowed participants to elaborate on their opinions extensively. We also checked with each participant that they had shared all their thoughts before ending the interview.

## Conclusion

Positive appraisal, easy availability, peer approval, time concerns, evidence, and PtDA awareness all affect whether health professionals are likely to use a PtDA to help pregnant women make decisions about screening for DS. These factors could inform the design of relevant PtDAs for DS screening and inform tailored strategies for implementing them. Our team will use these qualitative results to develop a theory-based survey questionnaire to quantitatively identify the determinants of health professionals’ intention to use a PtDA for DS screening decisions in preparation for an implementation intervention to promote its optimal use in routine clinical practice. In parallel, our team is leading a study with the same objectives with pregnant women [39].

## Additional file

Additional file 1: Frequency of all influential factors reported by each category of health professional and overall. *TDF* Theoretical Domains Framework, *#IF* Influential factor number (see Tables 2 and 3), *FP* family physicians, *MW* midwives, *OG* obstetrician-gynecologists, *Q* Number of quotes for each influential factor , *%QC* Percentage of times that each influential factor was repeated per construct. (XLSX 37 kb)

## Abbreviations

%QC: Percentage of times that each influential factor was repeated per construct
DS: Down syndrome
FP: Family physicians
IF: Influential factor
MW: Midwives
OG: Obstetrician-gynecologists
PtDAs: Patient decision aids
Q: Number of quotes for each influential factor

## References

[1] Lou S, Mikkelsen L, Hvidman L, Petersen OB, Nielsen CP (2015). Does screening for Down’s syndrome cause anxiety in pregnant women? A systematic review. Acta Obstet Gynecol Scand.

[2] St-Jacques S, Grenier S, Charland M, Forest JC, Rousseau F, Legare F (2008). Decisional needs assessment regarding Down syndrome prenatal testing: a systematic review of the perceptions of women, their partners and health professionals. Prenat Diagn.

[3] O’Connor AM, Drake ER, Wells GA, Tugwell P, Laupacis A, Elmslie T (2003). A survey of the decision-making needs of Canadians faced with complex health decisions. Health Expect.

[4] Woolf SH, Chan EC, Harris R, Sheridan SL, Braddock CH, Kaplan RM, Krist A, O’Connor AM, Tunis S (2005). Promoting informed choice: transforming health care to dispense knowledge for decision making. Ann Intern Med.

[5] Legare F, O’Connor AC, Graham I, Saucier D, Cote L, Cauchon M, Pare L (2006). Supporting patients facing difficult health care decisions: use of the Ottawa Decision Support Framework. Can Fam Physician.

[6] Chitty LS, Wright D, Hill M, Verhoef TI, Daley R, Lewis C, Mason S, McKay F, Jenkins L, Howarth A (2016). Uptake, outcomes, and costs of implementing non-invasive prenatal testing for Down’s syndrome into NHS maternity care: prospective cohort study in eight diverse maternity units. BMJ.

[7] Twiss P, Hill M, Daley R, Chitty LS (2014). Non-invasive prenatal testing for Down syndrome. Semin Fetal Neonatal Med.

[8] Silcock C, Liao LM, Hill M, Chitty LS (2015). Will the introduction of non-invasive prenatal testing for Down’s syndrome undermine informed choice?. Health Expect.

[9] Seror V, Ville Y (2010). Women’s attitudes to the successive decisions possibly involved in prenatal screening for Down syndrome: how consistent with their actual decisions?. Prenat Diagn.

[10] Legare F, St-Jacques S, Gagnon S, Njoya M, Brisson M, Fremont P, Rousseau F (2011). Prenatal screening for Down syndrome: a survey of willingness in women and family physicians to engage in shared decision-making. Prenat Diagn.

[11] Gagnon S, Labrecque M, Njoya M, Rousseau F, St-Jacques S, Legare F (2010). How much do family physicians involve pregnant women in decisions about prenatal screening for Down syndrome?. Prenat Diagn.

[12] Skjoth MM, Draborg E, Pedersen CD, Hansen HP, Lamont RF, Jorgensen JS (2015). Providing information about prenatal screening for Down syndrome: a systematic review. Acta Obstet Gynecol Scand.

[13] Makoul G, Clayman ML (2006). An integrative model of shared decision making in medical encounters. Patient Educ Couns.

[14] Charles C, Gafni A, Whelan T (1997). Shared decision-making in the medical encounter: what does it mean? (or it takes at least two to tango). Soc Sci Med.

[15] Bekker HL, Hewison J, Thornton JG (2003). Understanding why decision aids work: linking process with outcome. Patient Educ Couns.

[16] Neeman N, Isaac T, Leveille S, Dimonda C, Shin JY, Aronson MD, Freedman SD (2012). Improving doctor-patient communication in the outpatient setting using a facilitation tool: a preliminary study. Int J Qual Health Care.

[17] Stacey D, Legare F, Col NF, Bennett CL, Barry MJ, Eden KB, Holmes-Rovner M, Llewellyn-Thomas H, Lyddiatt A, Thomson R (2014). Decision aids for people facing health treatment or screening decisions. Cochrane Database Syst Rev.

[18] O’Connor AM, Graham ID, Visser A (2005). Implementing shared decision making in diverse health care systems: the role of patient decision aids. Patient Educ Couns.

[19] Legare F, Ratte S, Gravel K, Graham ID (2008). Barriers and facilitators to implementing shared decision-making in clinical practice: Update of a systematic review of health professionals’ perceptions. Patient Educ Couns.

[20] Joseph-Williams N, Newcombe R, Politi M, Durand MA, Sivell S, Stacey D, O’Connor A, Volk RJ, Edwards A, Bennett C (2013). Toward Minimum Standards for Certifying Patient Decision Aids: A Modified Delphi Consensus Process. Med Decis Making.

[21] Leiva Portocarrero ME, Garvelink MM, Becerra Perez MM, Giguere A, Robitaille H, Wilson BJ, Rousseau F, Legare F (2015). Decision aids that support decisions about prenatal testing for Down syndrome: an environmental scan. BMC Med Inform Decis Mak.

[22] Association pour la santé publique du Québec (ASPQ). Suivi de grossesse: quel professionnel choisir? Protégez-vous October 26, 2012.

[23] Godin G (2012). Les comportements dans le domaine de la santé: comprendre pour mieux intervenir.

[24] Giguere A, Legare F, Grad R, Pluye P, Haynes RB, Cauchon M, Rousseau F, Alvarez Argote J, Labrecque M (2012). Decision boxes for clinicians to support evidence-based practice and shared decision making: the user experience. Implement Sci.

[25] Michie S, Johnston M, Abraham C, Lawton R, Parker D, Walker A (2005). Making psychological theory useful for implementing evidence based practice: a consensus approach. Qual Saf Health Care.

[26] Huijg JM, Gebhardt WA, Dusseldorp E, Verheijden MW, van der Zouwe N, Middelkoop BJ, Crone MR (2014). Measuring determinants of implementation behavior: psychometric properties of a questionnaire based on the theoretical domains framework. Implement Sci.

[27] Francis JJ, Stockton C, Eccles MP, Johnston M, Cuthbertson BH, Grimshaw JM, Hyde C, Tinmouth A, Stanworth SJ (2009). Evidence-based selection of theories for designing behaviour change interventions: using methods based on theoretical construct domains to understand clinicians’ blood transfusion behaviour. Br J Health Psychol.

[28] Fishbein M, Hennessy M, Kamb M, Bolan GA, Hoxworth T, Iatesta M, Rhodes F, Zenilman JM (2001). Using intervention theory to model factors influencing behavior change. Project RESPECT. Eval Health Prof.

[29] Ministère de la santé et des services sociaux du Québec. Trisomy 21 prenatal screening program of Québec (pamphlet). [http://publications.msss.gouv.qc.ca/msss/fichiers/2015/15-931-01A.pdf]. Accessed 13 July 2016.

[30] Graham ID, Logan J, O’Connor A, Weeks KE, Aaron S, Cranney A, Dales R, Elmslie T, Hebert P, Jolly E (2003). A qualitative study of physicians’ perceptions of three decision aids. Patient Educ Couns.

[31] Graham ID, Logan J, Bennett CL, Presseau J, O’Connor AM, Mitchell SL, Tetroe JM, Cranney A, Hebert P, Aaron SD (2007). Physicians’ intentions and use of three patient decision aids. BMC Med Inform Decis Mak.

[32] Brace C, Schmocker S, Huang H, Victor JC, McLeod RS, Kennedy ED (2010). Physicians’ awareness and attitudes toward decision aids for patients with cancer. J Clin Oncol.

[33] Gravel K, Legare F, Graham ID (2006). Barriers and facilitators to implementing shared decision-making in clinical practice: a systematic review of health professionals’ perceptions. Implement Sci.

[34] Legare F, Stacey D, Briere N, Fraser K, Desroches S, Dumont S, Sales A, Puma C, Aube D (2013). Healthcare providers’ intentions to engage in an interprofessional approach to shared decision-making in home care programs: A mixed methods study. J Interprof Care.

[35] Couet N, Desroches S, Robitaille H, Vaillancourt H, Leblanc A, Turcotte S, Elwyn G, Legare F (2015). Assessments of the extent to which health-care providers involve patients in decision making: a systematic review of studies using the OPTION instrument. Health Expect.

[36] Informed Medical Decisions Foundation. http://www.informedmedicaldecisions.org/thoughtleadership/foundationblog/june-2012/at-the-state-policy-level,-shared-decision-making.aspx. June 19, 2012. Accessed 30 Aug 2016.

[37] Stacey D, Brière N, Robitaille H, Fraser K, Desroches S, Légaré F. A systematic process for creating and appraising clinical vignettes to illustrate interprofessional shared decision making. J Interprof Care. 2014;28(5):453–9. 10.3109/13561820.2014.911157 24766619

[38] Beenstock J, Sniehotta FF, White M, Bell R, Milne EM, Araujo-Soares V (2012). What helps and hinders midwives in engaging with pregnant women about stopping smoking? A cross-sectional survey of perceived implementation difficulties among midwives in the North East of England. Implement Sci.

[39] Delanoë A, Lépine J, Leiva Portocarrero ME, Robitaille H, Turcotte S, Lévesque I, Wilson BJ, Giguère AMC, Légaré F. Health literacy in pregnant women facing prenatal screening may explain their intention to use a patient decision aid: a short report. BMC Res Notes. 2016;9(1):339. 10.1186/s13104-016-2141-0 PMC4940686 27401163

